# Commentary: Understanding how informal dementia caregiver networks are assessed in the literature: results from a scoping review

**DOI:** 10.3389/frdem.2026.1970582

**Published:** 2026-09-28

**Authors:** Mi-Kyung Song, Sudeshna Paul, Mary Beth Happ

**Affiliations:** 1 Center for Nursing Excellence in Palliative Care, Nell Hodgson Woodruff School of Nursing, Emory University, Atlanta, GA, United States; 2 Nell Hodgson Woodruff School of Nursing, Emory University, Atlanta, GA, United States; 3 Golden Buckeye Center for Dementia Caregiving, The Ohio State University College of Nursing, Columbus, OH, United States

**Keywords:** caregiving-informal, dementia, measurement, social network analysis (SNA), standardization

Carlozzi et al. (2026) are to be commended for drawing attention to the considerable heterogeneity in how dementia caregiving networks are measured and reported. Their review highlights an important gap in the literature and underscores the need for greater consistency in caregiving-network assessment. We agree with this overarching conclusion. However, we contend that conceptual clarity regarding the nature and boundary of the caregiving network itself and what constitutes caregiving network data are a prerequisite for meaningful standardization.

Our concern is that the review identifies conceptual ambiguity as a major limitation of the literature while leaving several foundational questions about the network under study unresolved. Throughout the review, terms such as *informal dementia caregiver networks, caregiver networks, caregiving networks, care teams*, and *care team or network* are used, but it remains unclear whether these terms are intended to represent the same construct or distinct but overlapping phenomena. From a network-science perspective, this distinction is consequential because the specification of network boundaries, including who is included, who is excluded, who reports the network, and what constitutes a tie, is a fundamental requirement for network measurement and analysis (Laumann et al., 1983; Wasserman and Faust, 1994; McCarty et al., 2019). Different boundary specifications may produce substantially different representations of the same caregiving arrangement, limiting comparability across studies and complicating efforts toward standardization.

Relatedly, we question the distinction drawn between “caregiving networks” and “social networks.” The discussion appears to use “social networks” to refer to social support networks. However, in network science, social networks are broadly defined as actors connected through social relationships (Wasserman and Faust, 1994; Mitchell, 1969; Valente, 2010), and both social support networks and caregiving networks may be understood as specific forms of social networks. If the concern is that some studies included individuals providing social support rather than direct care, we agree this is an important issue but suggest framing it more explicitly as a question of network boundaries. Clarifying this distinction would improve conceptual precision.

A related issue concerns the lack of distinction between studies grounded in social network concepts and methods and network-oriented or multimember-oriented studies. We use the term, *network-oriented or multimember-oriented studies*, to refer to studies that assess caregiving beyond the primary caregiver but do not explicitly conceptualize caregiving as a network of actors connected through relational ties. Although those studies may address caregiving involving multiple caregivers, they do not study networks as the unit of analysis. Network studies use individual (node) level measurements and their direct local positions by which more detailed picture of the interrelationships (structure) is obtained (Wasserman and Faust, 1994; McCarty et al., 2019; Mitchell, 1969; Valente, 2010). The review synthesizes studies using social network analysis approaches alongside studies that assess the availability of helpers, caregiving hours, or satisfaction with support (Carlozzi et al., 2026; Song et al., 2023). By combining network-oriented or multimember-oriented studies with studies that explicitly examine relational structure, the review may obscure important differences in how caregiving networks have been conceptualized, operationalized, and studied.

This issue is reflected in the review's synthesis of caregiver-network measures. Variables such as network size and caregiver relationships are presented along with satisfaction with support and availability of help as common “caregiver network elements.” Yet, these constructs represent different levels of analysis, including network characteristics, perceptions of network functioning, and outcomes associated with networks. Further, we note an apparent inconsistency between the review's distinction between social support networks and caregiving networks and its discussion of measurement instruments. While studies focusing solely on social support networks were excluded, several measures highlighted as validated assessments to evaluate components of the caregiver networks were in fact originally developed to assess social support rather than caregiving-network structure, including the Inventory of Socially Supportive Behavior, Arizona Social Support Interview Schedule, Duke Social Support Index, and MacArthur Social Support Scale. Greater conceptual separation among these different levels of analysis and domains may help advance measurement development.

This lack of conceptual differentiation may also mask the relatively limited body of research that has explicitly used a social network approach. Among these studies, network domains consistently assessed include network size, composition, and members' caregiving roles. However, only one study employed formal network measures and analysis metrics (e.g., density and degree centrality) beyond the network size and composition (Song et al., 2023). Nonetheless, network boundary definitions and approaches to network member enumeration varied across studies.

The conceptual challenges identified in the review are not unique to dementia caregiving research. Similar questions regarding network boundaries, membership, relational ties, and network characterization have long been central concerns within social network science (Laumann et al., 1983; Wasserman and Faust, 1994; McCarty et al., 2019; Mitchell, 1969; Valente, 2010). We share the authors' view that the field would benefit from greater standardization of caregiving-network assessment. However, conceptual clarity regarding network boundaries and the distinction between network-oriented and network-science approaches may be necessary for standardization to be meaningful. We hope these observations contribute to ongoing discussion regarding the development of a more theoretically grounded science of caregiving networks.
